# An Assessment of the Profile and Predictors of Outcomes in COVID-19 Patients Hospitalized in a Tertiary Care Institute in Central India

**DOI:** 10.7759/cureus.26909

**Published:** 2022-07-16

**Authors:** Priyanka Parhad, Abhiruchi Galhotra, Atul Jindal, Nitin M Nagarkar, Ajoy K Behera

**Affiliations:** 1 Department of Public Health, All India Institute of Medical Sciences, Raipur, Raipur, IND; 2 Department of Community and Family Medicine, All India Institute of Medical Sciences, Raipur, Raipur, IND; 3 Department of Pediatrics, All India Institute of Medical Sciences, Raipur, Raipur, IND; 4 Department of Ear, Nose, and Throat, and Head and Neck Surgery, All India Institute of Medical Sciences, Raipur, Raipur, IND; 5 Department of Pulmonary Medicine and Tuberculosis, All India Institute of Medical Sciences, Raipur, Raipur, IND

**Keywords:** comorbidities, sars-cov-2, mortality, predictors, covid-19

## Abstract

Background

Coronavirus disease 2019 (COVID-19) is the largest pandemic that has affected people around the globe. Various researches have been conducted worldwide, but there is a scarcity of data from Central India on the relationship between several risk factors for infection and mortality. Our study assessed the predictors and patient profiles of those with COVID-19, which will aid in prioritizing patient treatment and preventive measures.

Methods

A retrospective study was done between March and December 2020. The study included 5,552 COVID-19 patients admitted to the All India Institute of Medical Sciences (AIIMS), Raipur. A validated questionnaire form provided by the WHO was used. Data for multiple clinical and nonclinical parameters were collected, and analysis was done using SPSS version 26 (IBM Corp., Armonk, NY, USA) and STATA version 12 (StataCorp LLC, College Station, TX, USA). Mortality and risk assessment of patients was done using multivariate logistic regression.

Result

In our study cohort of 5,552 COVID-19 patients, the median age was found to be 47 years (interquartile range (IQR): 31-60 years; range: 14-100 years), and 3,557 (64%) were male. Predominantly, patients presented with fever (41.30%), cough (40.20%), and dyspnea (29.29%). The major comorbidities were hypertension (29.70%), diabetes (25.40%), and chronic cardiac disease (5.79%). The common complications were liver dysfunction (26.83%), viral pneumonitis (23.66%), acute renal injury (15.25%), and acute respiratory distress syndrome (ARDS) (13.41%). In multivariate analysis, age (more than 40 years) (odds ratio (OR): 2.63; 95% confidence interval (CI): 1.531-4.512; p<0.001), diabetes (OR: 1.61; 95% CI: 1.088-2.399; p=0.017), obesity (OR: 6.88; 95% CI: 2.188-12.153; p=0.004), leukocytosis (OR: 1.74; 95% CI: 1.422-2.422; p<0.001), lymphocytopenia (OR: 2.54, 95% CI: 1.718-3.826; p<0.001), thrombocytopenia (OR: 1.15; 95% CI: 1.777-8.700; p=0.001), and ferritin concentration > 1,000 ng/mL (OR: 4.67; 95% CI: 1.991-10.975; p<0.001) were the independent predictors of mortality among COVID-19 patients.

Conclusion

The leading comorbidities in our study were hypertension, followed by diabetes. Patients who were 40 years or older, obese patients, and diabetic patients have a higher mortality risk. The poor prognostic predictors in COVID-19 patients were high ferritin levels (>1,000 ng/mL), leukocytosis, lymphocytopenia, and thrombocytopenia.

## Introduction

The new coronavirus, severe acute respiratory syndrome coronavirus 2 (SARS-CoV-2), is the etiologic agent of coronavirus disease 2019 (COVID-19), which has emerged as a global crisis. Coronavirus has had a massive global negative impact. Coronaviruses (CoVs) consist of severe acute respiratory syndrome coronavirus 1 (SARS-CoV-1), Middle East respiratory syndrome-related coronavirus (MERS-CoV), and severe acute respiratory syndrome coronavirus 2 (SARS-CoV-2) [1]. This major outbreak of disease triggered a global pandemic. In December 2019 and January 2020, the first instances of this virus were reported in Wuhan, China, which eventually spread to every corner of the globe [2]. It raised global concern as the WHO declared it a pandemic [3]. Coronaviruses (CoVs) are a vast family of zoonotic RNA viruses that are enclosed and single-stranded and can rapidly mutate and recombine, resulting in unique CoVs [4]. Symptoms and clinical signs vary from the common cold to severe respiratory disorders leading to multi-organ failure and even death [5]. Patients who are severely ill develop coinfections with bacteria and fungi [6].

This disease has spread globally, leading to 544,564,994 SARS-CoV-2 cases and 6,361,802 deaths worldwide with significant morbidity and mortality as of July 4, 2022 [7]. A developing nation with the second-highest populous country in the world reported its first case of COVID-19 on January 30, 2020. By now, India holds the second-highest SARS-CoV-2 cases globally [8]. It includes 43,518,564 COVID-19 cases and 525,223 deaths within the country [9]. As Central India catered for a large number of cases, Chhattisgarh counts 11,54,727 cases and 14,038 COVID-19 deaths as of July 4, 2022 [10].

Diabetes, hypertension, and cerebrovascular disease are the most prevalent comorbidities in COVID-19-infected individuals [11]. Furthermore, older patients with comorbidities and infection had a higher admission rate to the critical care unit and mortality from COVID-19 disease [12].

Observational studies performed in different regions help know the various features, manifestations, and broader disease perspectives for any newly emerging diseases. It is well known by now that comorbid conditions associated with COVID-19 have higher mortality and poor clinical outcome, but the prevalence, risk factors, treatment being used, outcomes, and differential effects of various comorbidities and COVID-19 are less known. Also, there are multiple studies that suggest that the underlying comorbidities might increase the risk of COVID-19 infection and mortality, but they do not provide robust evidence of the association due to less sample size, leading to low estimation precision.

There are limited data available from Central India regarding the association between various risk factors associated with COVID-19 infection and mortality. Broad epidemiological data are vital to understanding comorbidities in adults infected with COVID-19. Hence, this study attempts to elaborate on various determinants such as epidemiological characteristics, clinical features, underlying disease, laboratory parameters, complications, and treatment methods associated with COVID-19 infection and related outcomes. This is vital in the early detection of risk variables that could be effective in identifying those who require critical care. Also, this will extend efforts to improve outcomes with the analysis of essential variables.

This article was previously presented as an abstract at the European Society of Intensive Care Medicine (ESICM) LIVES Digital Annual Congress 2021 on October 3-6, 2021.

## Materials and methods

Study design and sample

The All India Institute of Medical Sciences (AIIMS), Raipur, Ethics Committee gave its approval to this retrospective observational study (letter number: 1489/IEC-AIIMSRPR/2021, dated February 16, 2021). The study was conducted at AIIMS, Raipur. The study sample consists of COVID-19 patients (mode of testing include RT-PCR, rapid antigen test, and TrueNAT) older than 14 years who were admitted to different wards (COVID-19 unit isolation wards, high-dependency units (HDUs), and intensive care units (ICUs)) between March 19 and December 31, 2020. The exclusion criteria were patients under the age of 14, patients with missing reports or details, patients leaving against medical advice (LAMA), and patients who were brought in dead.

Data collection

The clinical, laboratory, and radiological characteristics, demographics, epidemiological data, medical history, underlying comorbidities, treatment (antiviral therapy, corticosteroid therapy, and anticoagulant therapy), and outcomes were extracted from medical records using a WHO-validated case record form.

Statistical analysis

MS Excel (Microsoft Corp., Redmond, WA, USA) was used to enter and compile data. Frequencies and percentages were used to express categorical variables. Means, medians, and interquartile ranges (IQRs) were used to describe continuous variables. When the data had a normal distribution, the independent group Student's t-test was performed, and when they had a non-normal distribution, the Mann-Whitney U test was employed. To compare the proportions, the chi-square test was employed; when the data was small, Fischer's exact test was used. Univariate analysis and multivariate logistic regression were employed to determine death-related risk factors. A p-value less than 0.05 was used to determine statistical significance. For analysis, SPSS version 26 (IBM Corp., Armonk, NY, USA) and STATA version 12 (StataCorp LLC, College Station, TX, USA) were used.

## Results

Baseline characteristics

The study was completed on 5,552 COVID-19-positive patients. Table 1 represents the baseline characteristics of the study cohort with respect to outcome. There were 3,557 (64%) male patients, the male/female ratio was 1.78, and the median age of the patients was 47 years (IQR: 31-60 years; range: 14-100 years). Approximately 99% of the study cohort were living in the urban area, and 559 (10%) patients were non-survivors.

There were around 3,765 (67.81%) symptomatic COVID-19-positive patients. Fever (41.30%), cough (40.20%), and shortness of breath (29.29%) were the predominant symptoms. Vomiting, nausea, diarrhea, abdominal pain, and headache were the less common symptoms.

In comparison, non-survivors were significantly older (median: 60 years; IQR: 49-68 years) than survivors (median: 45 years; IQR: 30-60 years) (p<0.001). Male predominance was seen among non-survivors (3,127 (63%)) in comparison with survivors (430 (77%)) (p<0.001). Mortality had a significant association with a sore throat, fever, cough, nausea/vomiting, dyspnea, altered consciousness, and headache (all: p<0.05).

Oxygen saturation and SpO2 at admission were less than 90% in 1,057 (19%) patients. It was found to be significantly associated with mortality among COVID-19 patients (p<0.05).

Comorbidities

Hypertension (1,649 (29.70%)), diabetes (1,410 (25.40%)), and chronic cardiac diseases (322 (5.79%)) were the predominant comorbidities present in these patients. Mortality was significant in hypertension, diabetes, chronic lung disease, chronic kidney disease (CKD), chronic hematological disease, chronic neurological disorder, patients who were obese, chronic cardiac disease, and liver disease (all: p<0.05) (Table 1).

**Table 1:** Baseline characteristics of COVID-19 patients Data are presented as median (IQR) or N (%). *p<0.05 was considered significant. CKD: chronic kidney disease; IQR: interquartile range; N: number

Characteristics	Survivors (N (%)) (4,993 (90))	Non-survivors (N (%)) (559 (10))	Total (N (%)) (5,552 (100))	p-value	
					Age (median (IQR))	45 (30-60)	60 (49-68)	47 (31-60)	0.000*	
					<30	1,123 (22.49)	19 (3.39)	1,142 (20.56)		
30-39	952 (19.06)	37 (6.61)	989 (17.81)						
				40-49	726 (14.54)	84 (15.02)	810 (14.58)		
50-59	920 (18.42)	131 (23.43)	1,051 (18.93)						
				60-69	807 (16.16)	161 (28.80)	968 (17.43)		
70-79	366 (7.33)	97 (17.35)	463 (8.33)						
				>80	99 (1.98)	30 (5.36)	129 (2.32)		
Sex									
					Male	3,127 (62.63)	430 (76.92)	3,557 (64)		0.000*	
Female	1,866 (37.37)	129 (23.07)	1,995 (36)						
Address									
Urban	4,930 (98.7)	557 (99.6)	5,487 (98.8)	0.059					
Rural	63 (1.3)	2 (0.35)	65 (1.1)						
Outcome	4,993 (90)	559 (10)	5,552		
Symptoms					
					Symptomatic	3,235 (64.8)	530 (95)	3,765 (67.81)		
Fever	1,964 (39.34)	329 (58.86)	2,293 (41.30)	0.000*						
					Cough	1,940 (38.85)	292 (52.24)	2,232 (40.20)	0.000*	
Sore throat	680 (13.62)	41 (7.33)	721 (12.99)	0.000*						
					Runny nose	211 (4.23)	21 (3.76)	232 (4.18)	0.599	
Chest pain	166 (3.32)	22 (3.94)	188 (3.39)	0.449						
					Myalgia	242 (4.85)	28 (5.01)	270 (4.86)	0.866	
Fatigue	241 (4.83)	37 (6.62)	278 (5.01)	0.065						
					Shortness of breath (dyspnea)	1,203 (24.09)	423 (75.67)	1,626 (29.29)	0.000*	
Headache	230 (4.61)	9 (1.61)	239 (4.30)	0.001*						
					Altered consciousness	4 (0.08)	9 (1.61)	13 (0.23)	0.000*	
Seizure	8 (0.16)	3 (0.53)	11 (0.20)	0.057						
					Abdominal pain	62 (1.24)	11 (1.96)	73 (1.31)	0.15	
Vomiting/nausea	93 (1.86)	27 (4.83)	120 (2.16)	0.000*						
					Oxygen saturation (SpO2) at admission (%)					
>94	4,242 (85)	59 (10.55)	4,301 (77.46)	0.000*						
90-94	163 (3.26)	30 (5.36)	193 (3.47)		
<90	588 (11.77)	469 (84)	1,057 (19)		
Comorbidities					
					Hypertension	1,360 (27.24)	289 (52)	1,649 (29.70)	0.000*	
Diabetes	1,144 (22.91)	266 (48)	1,410 (25.40)	0.000*						
					Chronic cardiac disease	245 (5)	77 (14)	322 (5.79)	0.000*	
CKD	164 (3.28)	77 (14)	241 (4.34)	0.000*						
					Cancer	74 (1.48)	13 (2.33)	87 (1.57)	0.128	
Chronic lung disease	61 (1.3)	20 (3.5)	81 (1.45)	0.000*						
					Chronic neurological disorder	47 (0.94)	14 (2.50)	61 (1.10)	0.001*	
Chronic hematological disease	52 (1.04)	8 (1.43)	60 (1.08)	0.000*						
										Liver disease	36 (0.72)	17 (3.04)	53 (0.95)	0.000*	
					Obesity	11 (0.22)	6 (1.07)	17 (0.31)	0.001*						
Thyroid disorder	272 (5.45)	27 (4.83)	299 (5.39)	0.74						

Laboratory parameters

The laboratory values during admission are listed in Table 2. Lower hemoglobin values, leukocytosis, lymphopenia, neutropenia, and thrombocytopenia were found to be significantly associated with non-survivors as compared to survivors (all: p<0.05).

Higher rates of mortality were seen in patients with raised alanine transaminase, aspartate transaminase, and total bilirubin levels (all: p<0.05). Prothrombin time, international normalized ratio, and activated partial thromboplastin time were found significantly raised among non-survivors (all: p<0.05). Blood glucose, blood urea nitrogen, creatinine value, and potassium level were significantly raised among non-survivors (all: p<0.05). Simultaneously, ferritin levels, D-dimer, IL6, and C-reactive protein (CRP) were also significantly raised (all: p<0.05).

**Table 2:** Laboratory findings at the admission of COVID-19 patients Data are presented as median (IQR) or N (%). *p<0.05 was considered significant. Laboratory values were collected at admission. Student t-test and Mann-Whitney U test were applied. AST: aspartate transaminase; ALT: alanine transaminase; SGOT: serum glutamic-oxaloacetic transaminase; SGPT: serum glutamic-pyruvic transaminase; APTT: activated partial thromboplastin time; PT: prothrombin time; INR: international normalized ratio; LDH: lactate dehydrogenase; CRP: C-reactive protein; WBC: white blood cell

Parameters	Total		Survivors		Non-survivors		p-value
	Median	IQR	Median	IQR	Median	IQR	
Hemogram							
Hemoglobin (g/L or g/dL)	12.8	11.3-14.2	12.8	11.4-14.2	12.4	10.4-13.9	0.000*
WBC (×10^3^/uL)	6.9	5.3-9.3	6.7	5.2-8.7	11.69	7.49-16.5	0.000*
Lymphocyte count (%)	24.5	14.4-13.4	26	17-35	8	4.9-13.6	0.000*
Neutrophil count (cells/μL)	63.5	52.5-76	61.7	51.5-72.8	85.2	79.4-90	0.000*
Platelets (×10^3^/uL)	224	168-288	225	170-289	206	149.2-280.2	0.000*
Liver function test							
AST/SGOT	30	22-46	29	21-42	53	35-83	0.000*
ALT/SGPT	26	16-44	25	15-42	37	24.75-63	0.000*
Total bilirubin (mg/dL)	0.60	0.44-0.83	0.59	0.44-0.80	0.78	0.54-1.2	0.000*
APTT	29.1	26.9-32	29	26.8-31.6	32.1	28.1-36	0.000*
PT (seconds)	10.6	10-11.2	10.5	10-11.1	11.5	10.6-13.1	0.000*
INR	1	1-1.1	1	1-1.1	1.1	1-1.3	0.000*
Glucose (mg/dL)	98	88-125	98	87.7-122	205	122.25-323.25	0.000*
Kidney function test							
Blood urea nitrogen (mg/dL)	25	19-37	24	19-33	56	38-91	0.000*
LDH	482	366-657	468	358-611	847.23	406-995	0.000*
Creatinine (mg/dL)	1.1	0.9-1.27	1.08	0.90-1.2	1.22	1-1.8	0.000*
Sodium (mmol/L)	139	136-142	139	137-141	139	135-143	0.882
Potassium (mmol/L)	4.1	3.7-4.5	4.06	3.7-4.4	4.42	1.01-6.6	0.000*
Biomarkers							
CRP (mg/L)	10.7	3-55	8	2-40.89	105.1	58.01-172.8	0.000*
IL-6	24.3	4.8-105.3	6.8	2.2-678	60.1	11.5-148	0.035*
D-dimer	1.14	0.46-5.6	31.05	0.57	5.6	2.7-10.8	0.000*
Ferritin	203.8	80.2-477.3	291.30	173	814.1	446.7-1,475.3	0.000

Complications and treatment

The common complications were viral pneumonitis (1314 (23.66%)), acute respiratory distress syndrome (ARDS) (745 (13.41%)), liver dysfunction (1490 (26.83%)), acute renal injury (847 (15.25%)), coagulopathies (657 (11.83%)), hyperglycemia (468 (8.42%)), stroke (11 (0.19%)), and cardiac arrest (150 (2.7%)). Complications were more in non-survivors. The various treatment measures given to COVID-19 patients is presented in Table 3.

**Table 3:** Complications and treatment measures among COVID-19 patients Data are presented as N (%). ARDS: acute respiratory distress syndrome

Complications	Survivors (N (%)) (4,993 (90))	Non-survivors (N (%)) (559 (10))	Total (N (%)) (5,552 (100))
Viral pneumonitis	767 (15.36)	547 (97.85)	1,314 (23.66)
ARDS	221 (4.42)	524 (93.73)	745 (13.41)
Coagulation disorder	448 (8.97)	209 (37.38)	657 (11.83)
Acute renal injury	608 (12.17)	239 (42.75)	847 (15.25)
Liver dysfunction	1,145 (22.93)	345 (61.71)	1,490 (26.83)
Hyperglycemia	310 (6.20)	158 (28.26)	468 (8.42)
Stroke	5 (0.1)	6 (1.07)	11 (0.19)
Treatment			
Patients received oxygen therapy	900 (18.03)	546 (97.67)	1,446 (26.04)
Patients received mechanical ventilation	5 (0.02)	351 (62.79)	356 (6.41)
Patients received noninvasive ventilation	191 (3.83)	397 (71.02)	588 (10.59)
Antiviral treatment	839 (16.80)	268 (47.94)	1,107 (19.94)
Antibiotic treatment	4,303 (86.18)	529 (94.63)	4,832 (87.03)
Corticosteroid	602 (12.06)	191 (34.17)	793 (14.28)

Risk factors associated with case fatality

In univariate analysis, the risk factors linked to mortality at hospital admission were older age of more than 40 years; leukocytosis; lymphocytopenia; neutrophilia; thrombocytopenia; elevated AST, ALT, and APTT; and raised creatinine, CRP, and ferritin concentration. The presence of comorbidities such as hypertension, chronic cardiac diseases, diabetes, chronic lung diseases, chronic kidney diseases, liver diseases, chronic neurological disorders, and obesity was significantly associated with the case fatality rate (Table 4).

**Table 4:** Univariate analysis of variables associated with mortality in COVID-19 patients *p<0.05 was considered significant. AST: aspartate transaminase; ALT: alanine transaminase; APTT: activated partial thromboplastin time; CRP: C-reactive protein; PT: prothrombin time; WBC: white blood cell

Variables	Odds ratio	p-value	95% confidence interval	
				Age				
<40	1			
>40	6.38	0.000	4.818-8.466	
Sex				
Male	1			
Female	0.50	0.000	0.401-0.612	
Laboratory investigations				
Hemoglobin				
11-14	1			
<11	0.94	0.961	0.807-1.226	
>11	0.80	0.132	0.607-1.067	
WBC (×10^3^/uL)				
5-11	1			
<5	0.71	0.080	0.495-1.040	
>11	3.90	0.000	3.206-4.760	
Lymphocyte				
20%-40%	1			
<20%	11.17	0.000	11.101-21.469	
>40%	0.55	0.180	0.233-1.310	
Neutrophil				
40%-60%	1			
<40%	3.39	0.006	1.424-8.091	
>60%	19.84	0.000	11.803-33.367	
N/L ratio				
<3.5	1			
>3.5	1.31	0.720	0.297-5.791	
Platelet (×10^3^/uL)				
150-450	1			
<150	1.48	0.000	1.237-1.771	
>450	1.18	0.546	0.685-2.044	
AST				
<50	1			
>50	5.10	0.000	4.185-6.223	
ALT				
<50	1			
>50	2.25	0.000	1.829-2.769	
APTT				
<33	1			
>33	3.71	0.000	2.800-4.923	
Creatinine				
<1.3	1			
>1.3	3.07	0.000	2.562-3.697	
CRP				
<24	1			
>24	8.311	0.000	3.838-17.994	
Ferritin				
<500	1			
500-1,000	11.40	0.000	6.659-19.537	
>1,000	14.88	0.000	9.583-23.118	
Comorbidities				
Hypertension	2.85	0.000	2.394-3.413	
				Chronic cardiac diseases	3.09	0.000	2.357-4.066	
Diabetes	3.05	0.000	2.554-3.651	
				Chronic lung disease	3.00	0.000	1.796-5.009	
Chronic kidney disease	4.54	0.000	3.423-6.034	
				Liver diseases	4.31	0.000	2.409-7.741	
Chronic neurological disorder	2.70	0.001	1.478-4.941					
				Obesity	4.91	0.002	1.810-13.338	
Thyroid disorder	0.88	0.540	0.587-1.321					
				Cancer	1.44	0.224	0.798-2.610	

On multivariate analysis, age of more than 40 years, obesity, diabetes, leukocytosis, lymphocytopenia, thrombocytopenia, and increase in ferritin concentration (>1,000 ng/mL) were found to be significant independent predictors associated with mortality (Table 5).

**Table 5:** Multivariate analysis of risk factors associated with death among COVID-19 patients *p<0.05 was considered significant. APTT: activated partial thromboplastin time; AST: aspartate transaminase; CRP: C-reactive protein; WBC: white blood cell

Variables	Odds ratio	p-value	95% confidence interval
Age (years)			
<40	1		
>40	2.63	0.000	1.531-4.512
Sex			
Male	1		
Female	1.96	0.068	0.950-4.064
Comorbidities			
Hypertension	0.88	0.546	0.585-1.327
Chronic cardiac disease	0.85	0.752	0.315-2.300
Diabetes	1.61	0.017	1.088-2.399
Chronic lung disease	1.47	0.546	0.415-5.259
Chronic kidney disease	1.15	0.813	0.346-3.864
Liver disease	3.28	0.695	0.008-6.253
Obesity	6.88	0.004	2.188-12.153
WBC (×10^3^/uL)			
5-11	1		
<5	1.03	0.954	0.377-2.808
>11	1.74	0.000	1.422-2.422
Lymphocyte count			
20%-40%	1		
<20%	2.54	0.000	1.718-3.826
>40%	1.08	0.963	0.033-35.113
Neutrophil			
40%-60%	1		
<40%	2.53	0.085	0.644-8.529
>60%	4.16	0.211	0.444-38.971
Platelets			
150-450 (×10^3^/uL)	1		
<150	1.15	0.001	1.777-8.700
>450	1.04	0.944	0.282-3.893
Ferritin			
<500	1		
500-1,000	2.91	0.005	1.389-6.118
>1,000	4.67	0.000	1.991-10.975
CRP (mg/L)			
<24	1		
>24	1.63	0.090	0.952-2.234
APTT			
<33	1		
>33	2.35	0.060	1.254-4.429
AST			
<50	1		
>50	1.42	0.308	0.718-2.843

## Discussion

This study evaluated the demographic, clinical data, laboratory findings, complications, and mortality predictors among COVID-19 patients. Our sample consisted of 5,552 patients.

The median age was 47 years. The nationwide analysis by Guan et al. [13], analysis by Huang et al. in a Chinese population [14], and analysis by Tiwari et al. in Indian settings had similar findings [13-15]. In concert with the recent study by Wang et al., COVID-19 was more prevalent among adult males than females [16]. Around 60% of the patients were males [17]. In our study, only 1% of the population belonged to the rural area. At that point in time, given the COVID-19 infrastructure in the country, patients were being managed at the nearest health center, so this disparity existed, and most of the medical infrastructure, especially the tertiary centers, were concentrated in the cities and villages where people find it difficult to head for the treatment.

Similar results from other studies elicited the most prevalent symptoms present in our study group, such as fever, cough, and dyspnea, which are more or less identical to common viral infections [14,16]. In a study carried out by Jain et al., the high prevalence of dyspnea in non-survivors was also evident [17].

In our study, the prevailing comorbid conditions among COVID-19 patients were hypertension, diabetes, chronic diseases (kidney, lung, and liver), cancer, etc. Overall, our findings support those of Guan et al. [13] and Zhou et al. [18] who recently published research on the prevalence of comorbidities in COVID-19 patients. The three most prevalent comorbidities in our study were hypertension and diabetes, followed by chronic cardiac disease, which is concurrent with the systematic review and meta-analysis conducted by Yang et al. [19]. They were seen more frequently among non-survivors in our study. Research carried out by Wang et al. [16] and Jain et al. [17] had similar findings.

Several aberrant biomarkers that indicate organ dysfunction were discovered in this research. Lymphocytopenia and thrombocytopenia were significantly associated with non-survivors, which is consistent with the results of the study by Tian et al. [20]. The level of variables reflecting liver function (AST, ALT, and total bilirubin), kidney function (creatinine, urea, and potassium), coagulation (APTT, PT, and D-dimer), inflammation (LDH and CRP), and ferritin levels were significantly raised and associated with mortality. The study by Zhou et al. had almost similar findings [18].

Defects in cellular immune response and cytokine storm due to COVID-19 infection had played a role in the deranged immune system [21]. In a study conducted by Terpos et al., the independent predictors of mortality, such as leukocytosis, lymphocytopenia, thrombocytopenia, and ferritin levels, were similar to our findings [22]. According to a study by Lino et al., the degree of inflammation present upon the COVID-19 patient's admission, as measured by elevated ferritin levels (more than 1,873 ng/mL), is independently predictive of in-hospital mortality, which is concurrent with our findings [23].

Because lymphocytes have the ACE2 receptor on their surface, SARS-CoV-2 might infect those cells directly and cause them to lyse. Furthermore, the cytokine storm may promote lymphocyte apoptosis, leading to a decreased lymphocyte count [22]. SARS-CoV-2 infects the lungs and causes diffuse alveolar damage, trapping megakaryocytes and preventing platelet release, resulting in thrombocytopenia [24]. Increased ferritin levels in the systemic circulation are a double-edged sword in acute-phase response and inflammation. Serum ferritin plays an important role in the acute-phase response, but it is not primarily considered; no doubt, in various illnesses, its level can be exceedingly high, which might guide in stratifying patients who are at high risk [25].

The results reported here keep with current knowledge that the elderly with comorbid conditions are more susceptible to severe infection and mortality [26]. The association between chronic comorbidities and clinical outcomes was investigated in depth in this study. Comorbidities such as hypertension, chronic cardiac disease, diabetes, chronic lung diseases, chronic kidney diseases, liver diseases, and obesity had a significant association with mortality in univariate analysis. However, in the multivariate model, only obesity and diabetes were found to be independent predictors of mortality, which is consistent with the meta-analysis by Poly et al. [27].

Obesity (BMI ≥ 30 kg/m^2^) and COVID-19 are linked to lower blood oxygen saturation due to a lack of ventilation at the base of the lungs. It can augment resistance of the airway, resulting in an enhanced effort to breathe, and is associated with reduced functional capacity, pulmonary compliance, and expiratory reserve volume. A decreased diaphragmatic excursion can influence aeration in patients with central obesity in a supine position [17]. Other characteristics of low-grade inflammation caused by obesity include aberrant cytokine and adipokine production, as well as interferon repercussions in the impaired immune response [27].

Diseases that suppress the immune system, such as diabetes, cause metabolic inflammation, decreasing the defense mechanism of the body and leading to the invasion of pathogens. Defects in cellular immune response and cytokine storm may develop acute respiratory distress syndrome in diabetics and various disease complications, leading to mortality [28]. Also, diabetic patients have frequently been prescribed thiazolidinedione (oral hypoglycemic). The expression of ACE2 is increased by thiazolidinedione. As a result, enhanced ACE2 expression may facilitate SARS-CoV-2 internalization, thereby increasing the risk of acquiring the disease [29].

In the present study, common complications, such as ARDS, liver dysfunction, and acute renal injury, were prevalent in non-survivors, which is similar to the global research [30].

Our study illustrated that approximately one-fourth of the patients received antiviral treatment. More than half of the patients who received oxygen therapy or were admitted to ICU/HDUs received steroid therapy. More than 85% of the patients received antibiotic therapy [14]. The rationale for using antibiotics and antivirals is yet to be established. SARS-CoV-2 is a new virus, and no effective treatment has yet been identified. Organ support therapy is the cornerstone of SARS-CoV-2 infection treatment for critically ill patients.

The specific limitations that must be considered in our study include the retrospective design of the study; it is subjected to documentation errors. The patient's self-reported comorbidities might have underestimated the actual burden of comorbidities in the patients since some of them may be undiagnosed. Also, we considered only those COVID-19 patients hospitalized during the first wave of COVID-19; more research is required to compare morbidity and mortality during the first, second, and third waves of COVID-19. Lastly, we believe that our study cohort represented cases of COVID-19 in our state, Chhattisgarh, because we included all adult COVID-19 patients.

## Conclusions

The current study evaluated various risk factors linked to mortality among COVID-19 patients. Our findings strongly suggest that individuals older than 40 years had more than twice the mortality risk. Hypertension and diabetes were the main prevalent comorbidities. People with obesity and diabetes have a greater mortality risk. Furthermore, leukocytosis, thrombocytopenia, lymphocytopenia, and elevated ferritin concentration can also be regarded as unfavorable prognostic indicators in patients with COVID- 19. In critical patients, a comprehensive evaluation of the predictors can aid in classifying risk and ameliorating the prognosis.
